# Hydrogen Sulfide Deficiency Contributes to Tubular Damage and Calcium Oxalate Crystal Formation in Hyperoxaluria Nephropathy: Role of Osteopontin and Tamm–Horsfall Protein

**DOI:** 10.3390/antiox14091088

**Published:** 2025-09-05

**Authors:** Chien-Lin Lu, Yi-Shiou Tseng, Wen-Bin Wu, Chun-Hou Liao, Ming-Chieh Ma

**Affiliations:** 1 School of Medicine, College of Medicine, Fu Jen Catholic University, New Taipei 242062, Taiwan; 096195@mail.fju.edu.tw (C.-L.L.);; 2 Division of Nephrology, Department of Internal Medicine, Fu Jen Catholic University Hospital, Fu Jen Catholic University, New Taipei 243089, Taiwan; 3 Division of Urology, Department of Surgery, Far Eastern Memorial Hospital, New Taipei 220216, Taiwan; 4 Department of Chemical Engineering and Materials Science, Yuan Ze University, Taoyuan 320315, Taiwan; 5 Divisions of Urology, Department of Surgery, Cardinal Tien Hospital, New Taipei 231009, Taiwan

**Keywords:** hydrogen sulfide, hyperoxaluria, calcium oxalate, renal injury, specific protein 1, protein kinase A, anticrystallization molecule

## Abstract

Hydrogen sulfide (H_2_S) exerts regulatory functions in kidney diseases. However, its protective role against kidney stone formation remains unclear. Here, we demonstrate that hyperoxaluria or oxalate exposure impairs H_2_S formation, leading to tubular injury and calcium oxalate (CaOx) crystal deposition in both in vivo and in vitro models. In male rats fed 5% hydroxy-L-proline (HP), time-dependent increases in urinary supersaturation, tubular damage, and renal CaOx deposition were observed compared to controls. These changes were associated with the decreased expression of H_2_S-producing enzymes and elevated urinary secretion of osteopontin (OPN) and Tamm–Horsfall protein (THP). Notably, the protein level and activity of specificity protein 1 (Sp1), a transcription factor regulating these enzymes, were markedly decreased in HP-treated kidneys. Chronic supplementation with the H_2_S donor GYY4137 (GYY) significantly attenuated HP-induced tubular injury and CaOx deposition by reducing OPN and THP secretion. Consistent with in vivo results, H_2_S donors mitigated oxalate-induced tubular cell damage and CaOx formation in MDCK cells. Mechanistically, oxalate activated cyclic AMP/protein kinase A (PKA) signaling, which promoted OPN and THP secretion; these effects were eradicated by the PKA inhibitor H89 or GYY. These findings indicate that hyperoxaluria impairs Sp1 transcriptional activity, resulting in H_2_S deficiency and compromised anticrystallization defense in oxalate-induced tubulopathy.

## 1. Introduction

Consuming too much food that is rich in oxalate may lead to metabolic abnormality, induce hyperoxaluria, and thus cause kidney stones [1]. Nephrolithiasis, particularly calcium oxalate (CaOx) urolithiasis, poses a growing global health burden due to its high prevalence and recurrence rates [2,3]. Although acute interventions effectively remove stones, recurrence remains common without sustained lifestyle changes, such as adequate hydration and dietary oxalate restriction [4]. Recurrent CaOx stone formation is increasingly linked to chronic kidney disease (CKD) progression, although its underlying mechanisms remain poorly understood [5,6].

While urine supersaturation is a prerequisite for stone formation, crystal retention is tightly regulated by changes in the microenvironment in renal tubules, especially under oxidative stress [7]. Our previous studies demonstrated that vitamin E deficiency or L-arginine-induced nitrosative stress exacerbates tubular damage and promotes CaOx crystal deposition in hyperoxaluric kidneys [8,9]. Enhancing antioxidant capacity using vitamin E supplements preserves the protein content of osteopontin (OPN) and Tamm–Horsfall protein (THP) in renal tissues, reduces their urinary loss, and alleviates tubular damage and CaOx deposition [10]. OPN is expressed in the S3 segment of proximal tubules, distal tubules, and collecting ducts, and it can be upregulated by oxidative stress [8,11], while THP is constitutively synthesized in the thick ascending limb of Henle’s loop and secreted apically into the lumen [12]. Beyond its anticrystallization role, OPN functions as a survival factor for tubular cells; the genetic deletion or neutralization of OPN exacerbates tubular apoptosis in obstructive nephropathy [13]. Previous research on THP knockout mice has demonstrated that there is more functional and histological damage, inflammation, and tubular necrosis in kidneys suffering with ischemia/reperfusion (I/R) insult [14]. These findings clearly indicate the protective roles of OPN and THP in kidneys. As a secreted protein, the membrane-bound serine protease hepsin can cleave THP and is responsible for its secretion in Madin–Darby Canine Kidney (MDCK) cells [15]. In addition, vasopressin mediates protein kinase A (PKA) activation, which also stimulates THP secretion in the polarized MDCK cells [16]. Interestingly, PKA is known to regulate oxalate transport in intestinal epithelial cells, potentially contributing to hyperoxaluria [17]. After tubular secretion, OPN and THP are key macromolecules in urine that manipulate crystal adhesion, nucleation, and aggregation [18]. Although OPN may be upregulated to compensate for THP loss, this is insufficient to prevent crystal accumulation [11,19].

Hydrogen sulfide (H_2_S) is a gaseous signaling molecule with antioxidant and cytoprotective effects in kidney diseases [20,21]. Three main H_2_S-producing-enzymes, cystathionine β-synthase (CBS), cystathionine γ-lyase (CSE), and 3-mercaptopyruvate sulfurtransferase (3-MST), are responsible for H_2_S synthesis at a μM range and all present in kidneys [22,23,24]. The downstream signaling of H_2_S has been shown to protect kidneys against I/R, urinary tract obstruction, diabetes, hypertension, and 5/6 nephrectomy-induced uremia [25,26]. We previously showed that decreases in renal H_2_S-producing enzyme expression resulted in H_2_S deficiency; this was due to loss of upstream transcription factor specificity protein 1 (Sp1) activity [22,23]. An exogenous supplement of H_2_S by the given H_2_S donor GYY4137 (GYY) ameliorates functional insufficiency and tubular damage in both in vitro and in vivo uremic CKD models [22,23].

Concerning the antilithiatic effect of H_2_S, an in vitro study has shown that H_2_S donors such as sodium hydrosulfate (NaHS) can destabilize CaOx stone agglomeration by adjusting the pH and facilitating calcium complex with (thio)sulfate moieties [27]. In a CaOx rat model, the administration of allicin, a garlic-derived natural H_2_S donor, attenuates the accumulation of CaOx crystals by increasing connexin 43 expression in gap junctions, thereby preventing CaOx progression [28]. These data suggest that H_2_S donors possess potential in the treatment of kidney stones. However, the role of H_2_S in modulating OPN and THP expression and secretion under hyperoxaluric conditions remains unclear. We therefore hypothesized that H_2_S may protect kidneys against hyperoxaluria-induced tubular injury and CaOx crystal formation via PKA signaling in both in vitro and in vivo CaOx models, thereby preserving the function of OPN and THP in terms of anticrystallization effects.

## 2. Materials and Methods

### 2.1. Animal Model and Experimental Hyperoxaluria

Male Wistar rats weighing approximately 200 g were obtained from BioLASCO, the authorized distributor of Charles River Laboratories in Taiwan. All the experimental protocols were reviewed and approved by the Institutional Animal Care and Use Committee of Fu Jen Catholic University (approval no. A11085) and conducted in accordance with the Guide for the Care and Use of Laboratory Animals (National Academy Press, Washington, DC, USA, 2011). Animals were maintained under controlled-temperature conditions with a 9 h light (08:00–17:00) and 15 h dark cycle. The rats were randomly assigned to groups and treated for periods ranging from 3 to 28 days. The control animals received standard chow and tap water, whereas the experimental rats were fed chow supplemented with 5% hydroxy-L-proline (HP, *w*/*w*) to induce hyperoxaluria. A 3-day feeding period produced hyperoxaluria without renal calcium oxalate deposition, while 7–28 days of HP treatment resulted in varying degrees of CaOx crystal accumulation in the kidneys [29]. Throughout the study, all rats had unrestricted access to food and water. Before 24 h of urine collection, animals were acclimatized in metabolic cages for two days.

### 2.2. Preparation of a Mini-Osmotic Pump for Chronic H_2_S Donor Delivery

Mini-osmotic pumps (model 2004; Alzet, Cupertino, CA, USA) were pre-activated by incubation in saline at 37 °C for 4 h prior to implantation. Following anesthesia with sodium pentobarbital (60 mg/kg, i.p.), the pumps were implanted subcutaneously as described previously and according to the manufacturer’s instructions [22,29]. Each pump was filled with the H_2_S donor GYY4137 (Sigma-Aldrich, St. Louis, MO, USA) at a concentration of 0.2 M in phosphate-buffered saline (PBS, 0.1 M, pH 7.4) using a Hamilton syringe, ensuring that no air bubbles were introduced. The pumps delivered the solution at a constant rate for 28 days under sterile conditions. For control animals, pumps were filled with an equivalent volume of vehicle solution.

### 2.3. Metabolic Cage Study

Following the induction of hyperoxaluria or administration of treatment, rats were individually housed in metabolic cages with unrestricted access to food and water to monitor body weight, food consumption, and water intake, as well as to collect 24 h of urine samples, as previously described [22,29]. Urine was collected in tubes containing penicillin G (2000 IU) and streptomycin (2000 IU) to prevent bacterial growth. At the end of the collection period, animals were anesthetized with sodium pentobarbital (60 mg/kg, i.p.), and approximately 200 μL of blood was obtained from the left renal vein for H_2_S determination. Subsequently, the rats were perfused with cold PBS (0.01 M, pH 7.4) via the transcardiac route to flush out blood [29]. The kidneys were then harvested, weighed, sectioned, and either snap-frozen at −80 °C or fixed in paraformaldehyde for further analyses.

### 2.4. Urinalysis for Crystalluria

Daily urine output was determined by weight and is expressed in mL/day. After collection, urine samples were centrifuged at 620× *g* for 10 min at 4 °C. The supernatant was separated for subsequent biochemical analyses using commercial assay kits, while the pellet was dried in an oven at 60 °C for 48 h to determine the dry weight. Urinary calcium concentrations were measured with an electrolyte analyzer (Dri-Chem 3500i, Fuji, Tokyo, Japan) [29], and magnesium levels were assessed using a kit from BioAssay Systems (Hayward, CA, USA). The amounts of oxalate and citrate were quantified using commercial kits as previously reported [29]. The supersaturation of urine with respect to CaOx was estimated by calculating the AP(CaOx) index using the following formula: (4076 × calcium^0.9^ × oxalate^0.96^)/[(citrate + 0.015)^0.60^ × magnesium^0.55^ × urine volume^0.99^] [29]. All the urinalysis measurements were performed in duplicate.

### 2.5. CaOx Crystal Deposition in Rat Kidneys

Kidney tissue sections (5 μm thick) were prepared and stained with Pizzolato’s method as described previously [29]. In brief, sections were deparaffinized, rehydrated with distilled water, and then incubated with a mixture of 30% hydrogen peroxide and 5% silver nitrate (1 mL, pH 6.0). The slides were exposed to illumination from a 60 W incandescent lamp at a distance of 15 cm for 20 min. During development, the slides were rinsed several times with fresh staining solution to eliminate gas bubbles, followed by thorough washing with distilled water. Afterward, sections were counterstained with nuclear fast red, dehydrated, and mounted. The extent of calcium oxalate crystal deposition was semi-quantitatively graded from 0 (no deposits) to 3 (extensive deposits) by a pathologist blinded to the experimental groups.

### 2.6. Tissue Localization of H_2_S-Producing Enzymes

The renal expression of CBS, CSE, and 3-MST was examined by indirect immunofluorescence as previously described [22,29]. Briefly, kidneys were postfixed in 4% paraformaldehyde with 10% sucrose at 4 °C, embedded in O.C.T. compound (Tissue-Tek, Sakura Finetek, Torrance, CA, USA), and frozen at −20 °C. Cryosections (5 μm) were prepared using a cryostat (Microm, Heidelberg, Germany) and mounted on coated slides. After rehydration and washing with PBS (0.01 M, pH 7.4), the sections were processed with a tyramide signal amplification kit (PerkinElmer, Waltham, MA, USA). Non-specific binding was blocked with 5% skimmed milk in PBS for 1 h, followed by overnight incubation at 4 °C with primary antibodies against CSE (sc-374249, 1:1000), CBS (sc-67154, 1:1000), or 3-MST (sc-374326, 1:500) (Santa Cruz Biotechnology, Santa Cruz, CA, USA). The sections were then incubated for 1 h at room temperature with fluorescein-conjugated secondary antibodies. The nuclei were counterstained with DAPI, and images were captured using an inverted fluorescent microscope (Leica Microsystems GmbH, Wetzlar, Germany) equipped with an imaging analysis system (Diagnostic Instruments, Sterling Heights, MI, USA). The mean fluorescence intensity and positive area percentage were quantified using ImageJ v1.54 software (NIH, Bethesda, MD, USA).

### 2.7. Determination of Tubular Damage Markers, THP, and OPN in Urine

Urinary kidney injury molecule-1 (KIM-1), an indicator of tubular damage, was quantified using a commercial ELISA kit (MD Biosciences, Oakdale, MN, USA) following the manufacturer’s instructions. Urine samples were centrifuged at 2000× *g* for 15 min at 4 °C, and the resulting supernatants were stored at −80 °C until analysis. The levels of OPN and THP were measured in duplicate using ELISA kits from Abcam (Cambridge, MA, USA) according to the supplied protocols.

### 2.8. Renal Tubular Cell Culture and Drug Treatment

MDCK cells, of distal tubular origin, were employed as an in vitro model as previously described [30]. The cells were obtained from the Bioresource Collection and Research Center (Hsinchu, Taiwan), originally derived from ATCC line CL-101. The culture media and supplements were supplied by Thermo Scientific HyClone (South Logan, UT, USA). The cells were maintained in Medium 199 containing 3% fetal bovine serum, sodium bicarbonate (1.5 g/L), penicillin (10,000 U/mL), and streptomycin (10,000 μg/mL) at 37 °C in a humidified incubator with 5% CO_2_. Subculturing was performed every 3 days at confluence. For experiments, cells were seeded in 12-well plates and allowed to grow for 2 days. To assess OPN and THP secretion, cells were seeded onto semipermeable filter inserts (0.4 μm pore size; Corning Costar, Corning, NY, USA) and cultured for 4 days until polarized monolayers formed, as described previously [16]. On the day of treatment, 100 μL of culture medium from each well was collected and mixed with test reagents at the final concentrations indicated.

Oxalate toxicity was examined at 0.3 and 0.5 mM (dissolved in 0.01 M PBS, pH 7.4). The 0.5 mM dose was chosen for mechanistic studies of H_2_S, as it reflects the levels detected in 28-day hyperoxaluric rat urine. Chemical treatments were selected based on EC_50_/IC_50_ values: NaHS (100 μM) and GYY4137 (30 μM) as H_2_S donors, H89 (0.5 μM) as a PKA inhibitor, and hepIn-13 (1 μM; MedChem Express, Monmouth Junction, NJ, USA) as a hepsin blocker. Unless otherwise noted, all the reagents were obtained from Sigma-Aldrich (St. Louis, MO, USA). The cells were treated with each agent alone or in combination for 4, 8, or 24 h.

### 2.9. Evaluation of Cytotoxicity and Cell Viability

Cell injury was assessed by measuring lactate dehydrogenase (LDH) release into the culture medium using a cytotoxicity detection kit (Roche Applied Science, Mannheim, Germany). The absorbance of the reaction product was recorded at 492 nm with a standard ELISA plate reader, and the LDH activity was quantified against a Sigma-Aldrich LDH standard, as previously reported [23].

Cell viability was determined using the 3-(4,5)-dimethylthiazol-2-yl)-2,5-diphenyltetrazolium bromide (MTT) assay (Sigma-Aldrich). Briefly, cells were treated with oxalate alone or in combination with inhibitors for 48 h. After treatment, the medium was removed, and cells were rinsed twice with PBS (pH 7.4). MTT solution (5 mg/mL) was then added to each well, and the cells were incubated at 37 °C for 4 h. The resulting optical density (O.D.) was measured immediately, and cell viability is expressed as a percentage relative to control cells using the formula viable cells (%) = [100 × (treated O.D./control O.D.)], as previously described [23].

### 2.10. Kinetics of CaOx Formation in Culture System

Following exposure to oxalate, either alone or in combination with the H_2_S donors NaHS or GYY, for 4, 8, or 24 h, cultures were examined under an inverted phase-contrast microscope at 200× magnification (Leica Microsystems GmbH, Wetzlar, Germany). CaOx crystals were quantified and are expressed as the number of crystals per 100,000 μm^2^ of culture area.

### 2.11. Measurement of cAMP Levels and PKA Activity

The cAMP levels and PKA activity were assessed using a commercial kinase assay kit (ab138880 and ab139435, respectively, Abcam, Cambridge, UK) in accordance with the manufacturer’s protocol. MDCK cells were treated for 24 h with PBS, oxalate, or GYY alone, or in combination, as described above. After treatment, the cells were lysed in protein lysis buffer supplemented with a protease inhibitor cocktail (BioVision, Milpitas, CA, USA) and phenylmethylsulfonyl fluoride (Santa Cruz Biotechnology, Santa Cruz, CA, USA), followed by centrifugation at 13,000 rpm for 20 min. The protein concentrations were determined with a commercial protein assay kit (Bio-Rad, Hercules, CA, USA).

For cAMP determination, fluorescence signals generated by horseradish peroxidase–conjugated cAMP displacement were recorded using a plate reader (excitation at 540 nm, emission at 590 nm), with a calibration curve ranging from 0.3 nM to 10 μM prepared on the same day. For PKA activity, substrate-coated microtiter plates were activated with kinase dilution buffer, and aliquots containing 5 μg of lysate protein and ATP were incubated for 90 min at 30 °C. After removing unbound material, a phospho-specific substrate antibody was applied for 1 h at room temperature, followed by washing and incubation with HRP-conjugated anti-rabbit IgG (1:1000) for 30 min. Colorimetric detection was achieved using tetramethylbenzidine substrate, and the reaction was terminated with stop solution. The absorbance was measured at 450 nm using a microplate reader.

### 2.12. Cellular Distribution of OPN and THP

Following 24 h of treatment, cells were fixed with 4% paraformaldehyde and permeabilized in 0.1% Triton X-100 prepared in PBS. After quenching endogenous peroxidase activity, cells were incubated overnight at 4 °C with primary antibodies against OPN (sc-21742, 1:200) or THP (sc-20631, 1:500) (Santa Cruz Biotechnology). On the following day, cells were treated with HRP-conjugated secondary antibodies for 1 h at room temperature. Immunoreactive signals were amplified using a tyramide signal amplification kit (PerkinElmer) and visualized under an inverted fluorescence microscope, with fluorescein labeling for OPN and tetramethylrhodamine labeling for THP. The cell nuclei were counterstained with DAPI.

### 2.13. Determination of H_2_S Levels in Urine and Cell Culture Medium

Urine and culture medium samples were centrifuged at 4 °C to collect supernatants. An aliquot of 200 μL from each sample was used to measure the H_2_S concentrations according to the manufacturer’s protocol and previously published methods [22,23]. Measurements were performed at 25 °C for 5 min using an ion-selective electrode (Lazar Research Laboratories, Los Angeles, CA, USA) connected to a Fisher Accumet Model 10 pH meter (Fisher Scientific, Pittsburgh, PA, USA). Standard curves were generated with NaHS solutions ranging from 0.1 to 100 μM. Urinary H_2_S levels were normalized to the protein content in each sample and are expressed as nanomoles per milligram of protein.

### 2.14. Western Blot Analysis

Renal cortical tissue and cultured cells were fractionated into cytosolic and nuclear proteins using a commercial extraction kit (BioVision) according to the manufacturer’s protocol. The culture medium was collected and concentrated by centrifugation with ultrafiltration devices (Microcon, Millipore, Bedford, MA, USA). The protein concentrations were determined with a commercial assay kit (Bio-Rad). Equal amounts of protein were resolved on SDS–polyacrylamide gels under denaturing conditions and transferred to polyvinylidene difluoride membranes (Amersham-Pharmacia Biotech, Little Chalfont, UK). After blocking with 5% skim milk, the membranes were incubated overnight at 4 °C with primary antibodies against CBS (1:1000), CSE (1:2000), 3-MST (1:500), Sp1 (sc-17824, 1:1000), OPN (1:500), THP (1:500), β-actin (1:2000), or histone (1:500) (Santa Cruz Biotechnology). After washing, the membranes were probed with the appropriate HRP-conjugated IgG (Jackson ImmunoResearch, West Grove, PA, USA) for 1 h at room temperature. Antibody binding was visualized with an enhanced chemiluminescence kit (Thermo Scientific, Rockford, IL, USA). The intensities of bands corresponding to the expected molecular weights were quantified semi-quantitatively by densitometry using an image analysis system (Diagnostic Instruments, Sterling Heights, MI, USA). The protein expression levels were normalized to β-actin or histone as internal controls.

### 2.15. Determination of Sp1 Activity

Nuclear protein extracts were obtained from renal tissues and cultured tubular cells using a commercial extraction kit (BioVision), following the manufacturer’s protocol. Sp1 activity was quantified with a high-throughput assay kit (TFAB00160, Assay Genie, Dublin, Ireland). In this assay, a double-stranded DNA sequence containing the Sp1 consensus binding site was immobilized on a 96-well plate. Active Sp1 in the nuclear extracts bound specifically to this sequence and was detected by a primary antibody recognizing the Sp1 epitope. Thus, only DNA-bound (active) Sp1 was measured. Detection was achieved using an HRP-conjugated secondary antibody, and the absorbance was read at 450 nm.

### 2.16. Quantitative Real-Time PCR for H_2_S-Producing Enzyme Expression

Total RNA was isolated using a commercial extraction kit (RareRNA, Bio-East Technology, Taipei, Taiwan) as previously described [23]. Complementary DNA (cDNA) was synthesized at 42 °C for 45 min from 2 μg of RNA using 5 μg of poly(dT)_15_ primer (Life Technologies, Waltham, MA, USA) and 200 units of Moloney murine leukemia virus reverse transcriptase (Promega, Madison, WI, USA). Quantitative real-time PCR was carried out with an ABI StepOne Plus system (Applied Biosystems, Foster City, CA, USA). The reactions (20 μL) contained 100 ng of cDNA, 30 μM primers, and SYBR Green PCR master mix (Applied Biosystems). The primer sequences are listed in Table 1 [23]. The amplification protocol consisted of an initial denaturation at 95 °C for 20 s, followed by 40 cycles of 95 °C for 1 s and 60 °C for 20 s. A melting curve analysis was performed at the end of amplification to confirm specificity. All the reactions were conducted in duplicate. The relative gene expression was calculated using the ΔCt method, where ΔCt was defined as the difference between the Ct value of the target gene and that of GAPDH. Fold changes in mRNA levels of H_2_S-producing enzymes were expressed relative to the control group using the 2^−ΔCt^ method.

### 2.17. Statistics

All the numerical data are expressed as the mean ± standard deviation (S.D.). Group comparisons were performed using either an unpaired Student’s *t*-test or one-way ANOVA followed by Duncan’s multiple range post hoc test. A *p*-value < 0.05 was considered statistically significant.

## 3. Results

### 3.1. Hyperoxaluria Induces Kidney Injury, CaOx Crystal Deposition, and H_2_S Deficiency

**Figure 1:**
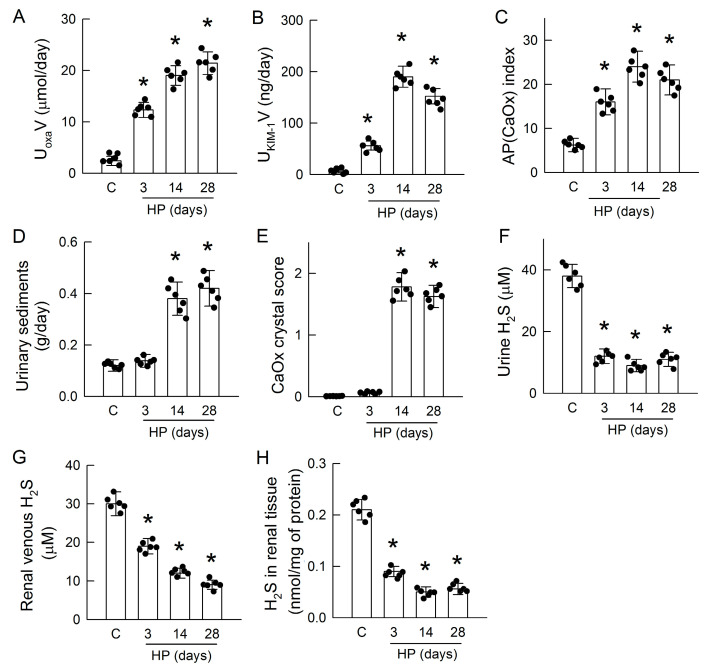
Effect of hyperoxaluria on kidney injury, CaOx crystal formation, and H_2_S levels. (**A**) Daily urine samples obtained from a metabolic cage study showing hyperoxaluria after treatment with hydroxy-L-proline (HP). Control (C) urine was collected only on day 28. HP-induced hyperoxaluria showed a progressive increase over time. (**B**) Urinary supersaturation was estimated using the AP(CaOx) index and increased at all the time points of induction. (**C**) Urinary kidney injury molecule-1 (KIM-1), a marker of tubular injury, increased significantly in HP-treated rats throughout the induction period. (**D**) HP increased urinary sediments after 14 and 28 days of induction through enhanced CaOx excretion. (**E**) Hyperoxaluria increased renal CaOx crystal formation and deposition on days 14 and 28 after scoring. (**F**) H_2_S was measured in urine after collection from the metabolic cage. (**G**) H_2_S was determined in blood sampled from the renal vein. (**H**) H_2_S was measured in the renal tissue of the groups. The scatter dots in the bar graphs show the data distribution. N = 6 in each group. * *p* < 0.05, HP versus control.

In rats fed with an HP diet, we showed a successful induction of hyperoxaluria (Figure 1A), which is associated with increases in the urinary levels of KIM-1 (Figure 1B); the degree of urine supersaturation as an ion activity product of CaOx [AP(CaOx)] index (Figure 1C) increased in a time-dependent manner from 3 to 28 days. Some peak increases in these parameters were found 14 days after induction. Compared with the control rats, urinary excretion of CaOx crystals represented by the dry weight of urine sediment (Figure 1D) and intrarenal CaOx crystal deposition (Figure 1E) increased on day 14 and 28, but not on day 3 after hyperoxaluric induction. Interestingly, these changes were associated with impaired H_2_S formation, as evidenced by a low urinary excretion of H_2_S (Figure 1F), as well as reductions in H_2_S levels in renal venous blood (Figure 1G) and in kidney tissues (Figure 1H) at all time points in HP rats.

### 3.2. Hyperoxaluria Enhances Renal OPN and THP Excretion and Decreases Sp1 Activity

**Figure 2:**
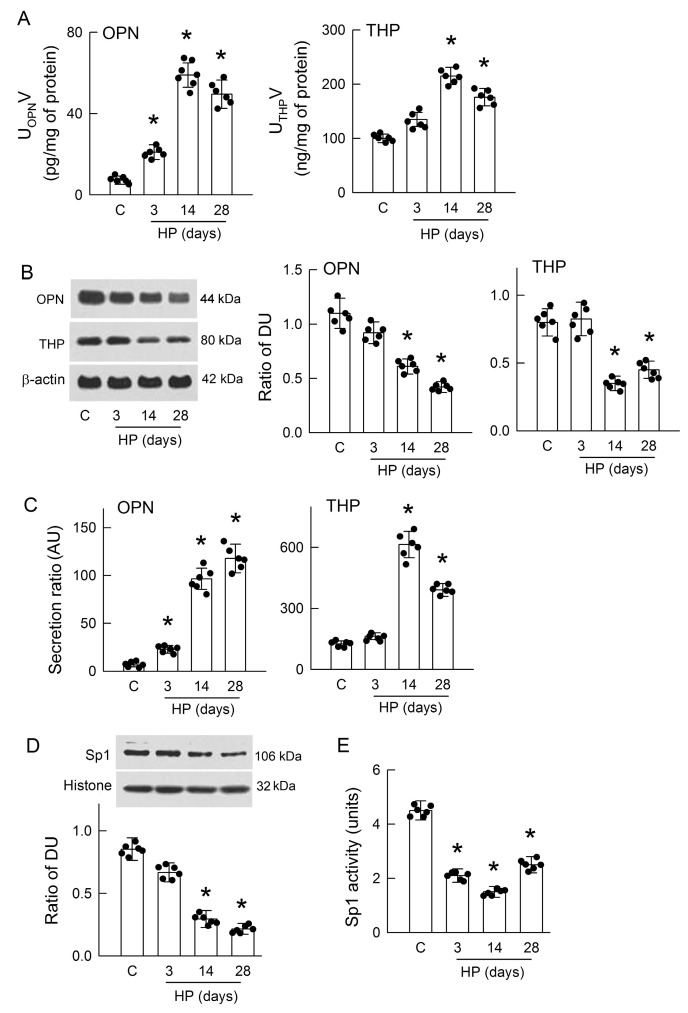
Hyperoxaluria-induced increases in renal osteopontin (OPN) and Tamm–Horsfall protein (THP) excretion and decreases in specificity protein 1 (Sp1) function. (**A**) The amounts of OPN (**left**) and THP (**right**) in urine after 24 h of collection from metabolic cage for control (C) and HP rats were determined using ELISA kits. (**B**) Representative immunoblots of renal OPN and THP expression, with β-actin as an internal control. The right two bar graphs display the quantification of band intensity, calculated as density units (DU) normalized to β-actin. (**C**) The secretion ratios of OPN and THP in urine were estimated by dividing the protein expression in the kidney and are expressed as arbitrary units (AU). (**D**) Western blot analysis of Sp1 and β-actin in kidney tissue, with the lower panel showing the quantification of Sp1 relative to β-actin. (**E**) The protein activity of Sp1 in control and HP kidneys at various time points was examined. The scatter dots in the bar graphs show the data distribution. N = 6 in each group. * *p* < 0.05, HP versus control.

OPN and THP are the major urine proteins and play a role in kidney stone formation [31,32]. Our results showed that urinary excretion of OPN and THP was increased in the HP kidneys when compared to the control group, with peak increases on day 14 (Figure 2A). On day 3 of hyperoxaluria, urinary THP excretion was increased but this increment did not reach statistical significance.

As renal tubules are the dominant tissue for OPN and THP formation and release [32], we tested whether hyperoxaluria may affect the renal expression of OPN and THP. Our results showed that OPN and THP were significantly decreased at 14 and 28 days in the HP kidneys when compared to the control kidney (Figure 2B). In the HP kidneys with 3 days of treatment, OPN expression was slightly low but insignificant. We then estimated their secretion by dividing urinary amounts of OPN and THP by their intrarenal protein expressions. Interestingly, our results showed that renal OPN secretion was time-dependent for increases in the HP kidneys (Figure 2C). Increases in renal THP secretion were found in the 14- and 28-day HP kidneys, with a peak increase on day 14. On day 3, THP secretion was slightly elevated but insignificantly so. These results indicate that enhanced OPN and THP secretion by hyperoxaluria probably contributes to CaOx crystal formation.

Due to the upstream function of transcription factor Sp1 on regulating H_2_S-producing enzyme expression [23], we investigated whether hyperoxaluria affects Sp1 activity. Compared with that in the control rats, the renal expression of Sp1 was markedly decreased in the HP rats on days 14 and 28 (Figure 2D). This was associated with low Sp1 activity in the renal tissues of the HP rats at all induction times when compared with that in the controls (Figure 2E). The parallel changes in Sp1 activity and H_2_S-producing enzyme expression clearly indicate the molecular interactions among these proteins.

### 3.3. Hyperoxaluria Is Harmful for H_2_S-Producing Enzyme Expression

**Figure 3:**
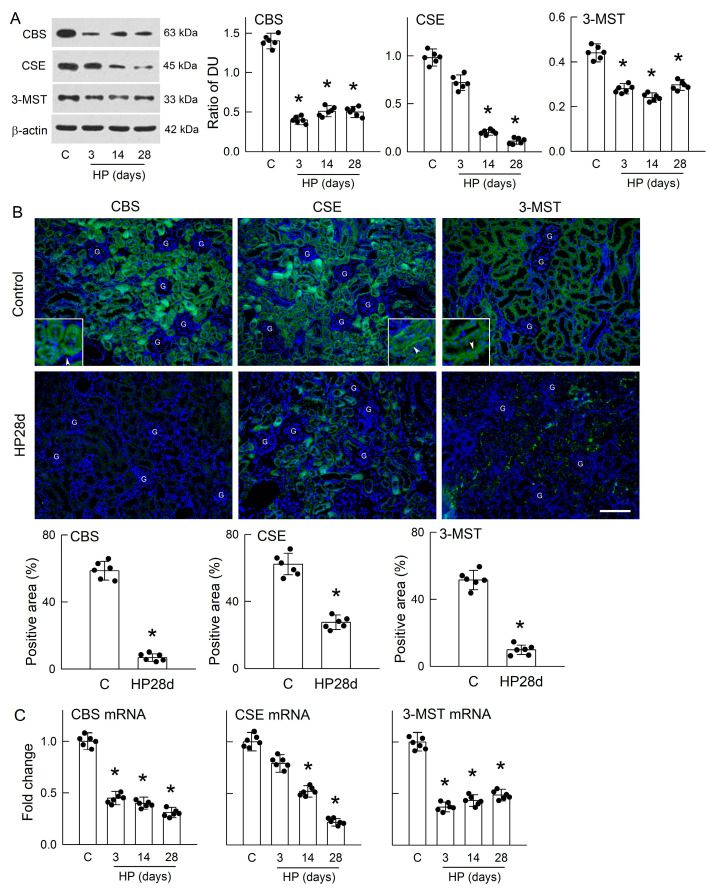
Hyperoxaluria decreases H_2_S-producing enzyme expression. (**A**) Representative immunoblots display the protein expression levels of the three main H_2_S-producing enzymes—cystathionine β-synthase (CBS), cystathionine γ-lyase (CSE), and 3-mercaptopyruvate sulfurtransferase (3-MST)—in renal tissues from control (C) and HP-treated rats over a 3–28-day period. The quantification shown in the right bar graphs represents density units (DU) normalized to β-actin. (**B**) In the rat renal cortex, the representative images show immunoreactivity (green color) for CBS, CSE, and 3-MST in the control (upper three) and HP (lower three) kidney after 28 days of treatment. Nuclei were counterstained using 4′,6-diamidino-2-phenylindole (blue color). Insets in the control kidney indicate more details for the tubular distribution of H_2_S-producing enzymes. Arrowheads indicate the intracellular distribution of CBS, CSE, and 3-MST in renal tubules. G, glomerulus. The white horizontal bar = 120 μm. The lower bar graphs represent the percentage of positive areas in each group after quantitation. (**C**) The mRNA levels of CBS, CSE, and 3-MST were measured in renal tissues at indicated time points using RT-qPCR, with the results expressed as fold changes relative to the control group. Each bar graph includes scatter dots representing individual sample values. N = 6 in each group. * *p* < 0.05, HP versus control.

Low Sp1 protein expression and activity led us to hypothesize that it might impair the function of H_2_S-producing enzymes. We then examined the intrarenal expression of three main H_2_S-producing enzymes. Compared with the controls, the protein levels of CBS and 3-MST were significantly reduced in the HP kidneys at all time points of induction (Figure 3A). For CSE, a significant decrease in protein levels was found only after 14 and 28 days of HP treatment.

The majority of CBS expression in the control kidneys was found in the tubular epithelium and the lining of the capillary of the renal cortex (upper right part of Figure 3B). Tubular expression of CBS was homogenously distributed in the cytoplasm of epithelial cells (inset in upper right picture). Less frequent distributions of CBS were found in glomeruli. In the HP kidney, the tubular expression of CBS was markedly decreased (lower right part of Figure 3B). Most of the CSE in the control kidneys was also present in the renal tubules, with a less frequent distribution in the capillaries and glomeruli (upper middle part of Figure 3B). The distribution of CSE in the renal tubular epithelium is close to the basolateral membrane (inset in upper middle part). Similarly to CBS, the tubular expression of CSE was greatly reduced in the HP kidneys (lower middle part of Figure 3B). The 3-MST was mainly expressed in the renal tubules of the control kidneys, with low expression in capillaries and glomeruli (upper left part of Figure 3B). Similarly to CSE, its expression is also close to the basolateral membrane of tubular cells (inset in upper left picture). The tubular expression of 3-MST was markedly attenuated in the HP kidneys (lower left part of Figure 3B). After quantification, 28 days of HP treatment led to significant decreases in the renal expression of all H_2_S-producing enzymes (see the bar graphs in Figure 3B).

Low protein expression of CBS, CSE, and 3-MST was associated with decreases in mRNA levels in the HP kidney when compared to those in the control kidney (Figure 3C). A decrease in CSE mRNA expression was observed on day 3; this, however, was statistically insignificant. These results clearly indicated that hyperoxaluria markedly reduced intrarenal H_2_S-producing enzyme expression at both the protein and mRNA levels.

### 3.4. Chronic H_2_S Supplementation Ameliorates Hyperoxaluria-Induced Tubular Damage, CaOx Deposition, and the Secretion of OPN and THP

**Figure 4:**
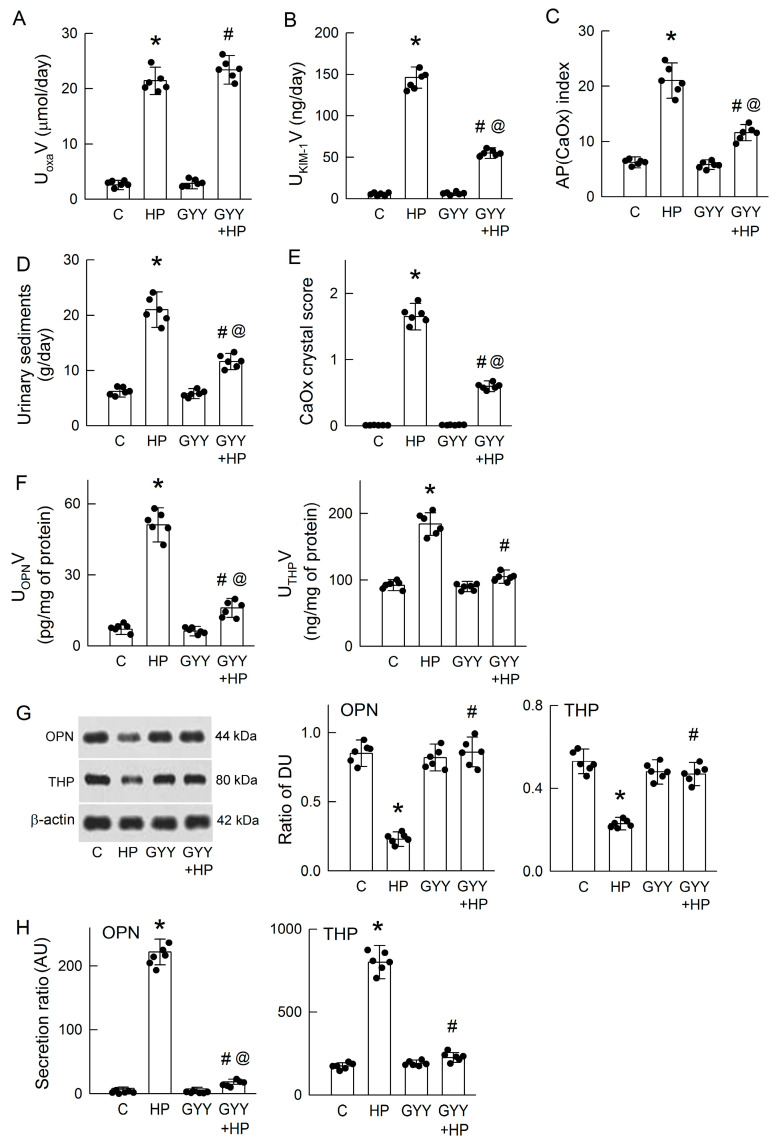
Chronic H_2_S supplementation attenuates hyperoxaluria-induced renal damage, CaOx formation, and OPN and THP secretion. (**A**) The H_2_S donor GYY4137 (GYY) does not affect increased urinary excretion of oxalate (U_oxa_V) in the 28-day HP kidney. (**B**) Urinary levels of the renal damage marker KIM-1 are determined and calculated as the excretion rate (U_KIM-1_V). (**C**) The degree of supersaturation in urine is estimated according to the AP(CaOx) index. (**D**) The precipitated particles in urine are collected, and CaOx crystal formation is evaluated according to the dry weight of the urinary sediment. (**E**) Renal CaOx crystal formation and deposition caused by hyperoxaluria, which is examined and scored after staining. (**F**) Urinary levels of OPN and THP are quantitated using ELISA kits. (**G**) The representative blots of one experiment show the protein expressions of OPN, THP, and β-actin in renal tissues. The right two bar graphs show the ratio of the band density unit (DU) of OPN or THP to β-actin. (**H**) The secretion ratios of OPN and THP in urine were estimated by dividing the protein expression in the kidney and expressed as arbitrary units (AU). Note that GYY reduces HP-induced increase in urinary OPN and THP secretion. The scatter dots in the bar graphs show the data distribution. N = 6 in each group. * *p* < 0.05, HP vs. control (C). ^#^ *p* < 0.05, GYY + HP vs. HP. ^@^ *p* < 0.05, GYY + HP vs. GYY.

Because H_2_S deficiency is associated with excess OPN and THP secretion in HP kidneys, we then tested whether exogenous H_2_S supplementation could reverse these changes and mitigate hyperoxaluria-induced CaOx crystal formation. Chronic administration of the H_2_S donor GYY for 28 days via a subcutaneous mini-osmotic pump did not affect the degree of hyperoxaluria (Figure 4A) but significantly attenuated kidney injury, as evidenced by reduced urinary KIM-1 levels (Figure 4B), along with decreases in urinary supersaturation (Figure 4C), the weight of urine sediments (Figure 4D), and intrarenal CaOx crystal deposition by scoring (Figure 4E). Interestingly, the hyperoxaluria-induced increases in urinary OPN and THP levels was markedly attenuated in the GYY-treated HP kidneys (Figure 4F). Moreover, GYY completely reversed the effect of hyperoxaluria on the attenuation of renal OPN and THP expression when compared to the control kidney. GYY also significantly attenuated the hyperoxaluria-enhanced tubular secretion of OPN and THP in HP kidneys (Figure 4H). However, GYY alone showed no effect on these parameters. These results clearly indicate that exogenous H_2_S supplementation protects the kidney against hyperoxaluric insult by reducing OPN and THP secretion for CaOx crystal formation and deposition.

### 3.5. Oxalate-Induced Cytotoxicity and CaOx Formation in Renal Tubular Cells

**Figure 5:**
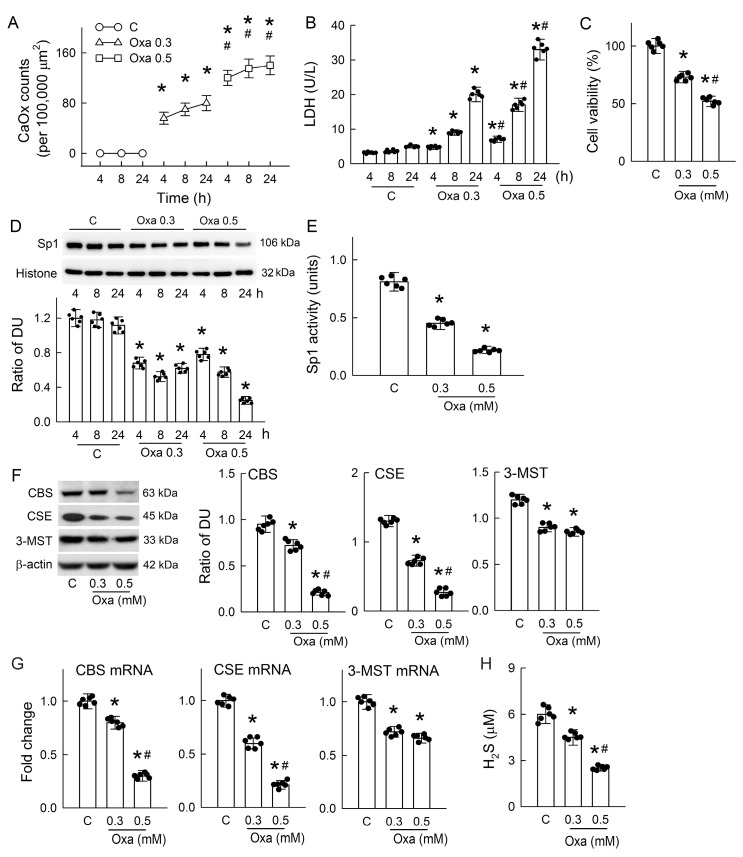
Oxalate induces calcium oxalate (CaOx) crystal formation, tubular cell injury, and H_2_S deficiency in MDCK cells. (**A**) Kinetics of CaOx crystal formation in the culture system after 4, 8, and 24 h of treatment with phosphate-buffered saline (PBS) as the control (C) group or with oxalate (Oxa, 0.3 and 0.5 mM). The CaOx crystals were counted and are expressed as numbers per area. Note the dose- and time-dependent effects of oxalate on CaOx crystal formation. (**B**) The lactate dehydrogenase (LDH) release was measured in a culture medium as the marker of cell damage. (**C**) After 8 h, the cell viability was determined via an MTT assay. (**D**) Representative immunoblots display Sp1 and histone expression following PBS or oxalate treatment across different doses and time points. The corresponding bar graphs below show the Sp1/histone ratio, presented in density units (DU). (**E**) Sp1 transcriptional activity was analyzed after 24 h of treatment with or without oxalate. (**F**) Immunoblot analysis of H_2_S-generating enzymes (CBS, CSE, and 3-MST) and β-actin after 8-h exposure to PBS or oxalate. The right-side bar graphs depict the quantification of each enzyme relative to β-actin. (**G**) The levels of CBS, CSE, and 3-MST mRNA were measured via RT-qPCR after 24 h of treatment and are expressed as fold changes compared to control. (**H**) H_2_S levels in the culture medium were measured in groups. Each bar graph includes scatter dots representing individual replicate data (N = 6 per condition). * *p* < 0.05, oxalate vs. control at the same time point. ^#^ *p* < 0.05, Oxa 0.5 vs. Oxa 0.3 at corresponding time point.

Our in vivo observations clearly indicated the harmful effect of hyperoxaluria on H_2_S production, and this deficiency contributes to CaOx crystal formation via OPN and THP. We then tested the in vitro effect of oxalate in renal tubular MDCK cells to see how H_2_S affects OPN and THP secretion during CaOx crystal formation. Oxalate treatment dose- and time-dependently increased CaOx crystal deposition in the culture system (Figure 5A). This was associated with tubulotoxicity, as indicated by increases in LDH release (Figure 5B) and attenuated cell viability (Figure 5C).

To understand the role of H_2_S, we first investigated the function of Sp1 because it can regulate the expression of H_2_S-producing enzymes [23]. Oxalate significantly decreased Sp1 expression at both concentrations compared to the control group; a time-dependent decrease was observed following treatment with 0.5 mM oxalate (Figure 5D). The protein activity of Sp1 was dose-dependently attenuated by oxalate (Figure 5E). Oxalate reduced the protein expression and mRNA levels of CBS, CSE, and 3-MST in a dose-dependent manner (Figure 5F,G). Interestingly, H_2_S release into the culture medium was also dose-dependently attenuated by oxalate (Figure 5H). These results indicate that oxalate-mediated H_2_S deficiency occurs due to impaired Sp1/H_2_S-producing enzyme function.

### 3.6. Oxalate Increases OPN and THP Secretion and Activates Protein Kinase A (PKA)

**Figure 6:**
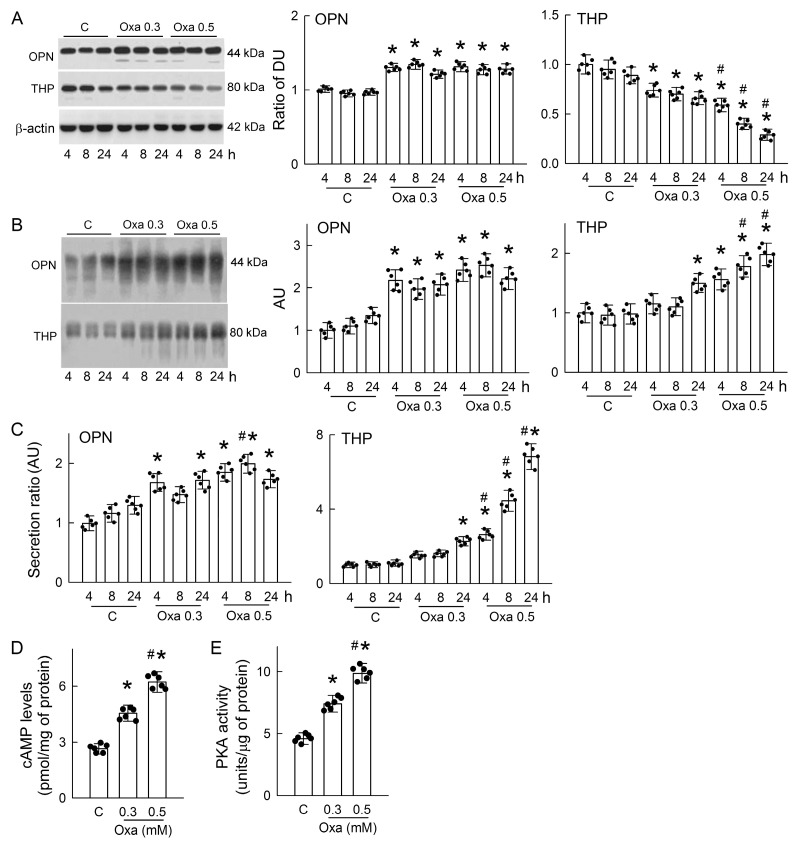
Oxalate increases OPN and THP secretion and activates PKA in cells. (**A**) The representative blots of one experiment show OPN, THP, and β-actin expression in MDCK cells after treatment with PBS (control, C) or oxalate (0.3 or 0.5 mM) for 4, 8, 24 h. The right bar graphs show the ratio of the band density (DU) of OPN or THP to β-actin. (**B**) The representative blots of one experiment show OPN and THP expression in the culture medium after cells treated with PBS or oxalate. The right bar graphs show the band density unit of OPN or THP and are expressed as the arbitrary unit (AU). (**C**) The secretion ratio of OPN and THP in the culture medium were estimated by dividing the protein expression in cells and are expressed as the AU. (**D**) The cAMP level was determined in cells after treatment with PBS or oxalate (0.3 or 0.5 mM) for 24 h. (**E**) The PKA activity was determined in cells after treatment with PBS or oxalate (0.3 or 0.5 mM) for 24 h. N = 6 experiments performed at each time point and dose. The scatter dots in the bar graphs show the data distribution. * *p* < 0.05, oxalate vs. control at the same time point. ^#^ *p* < 0.05, Oxa 0.5 vs. Oxa 0.3 at the same time point.

We then examined the direct effect of oxalate on OPN and THP secretion in MDCK cells as observed in our hyperoxaluric rats. Treatments of 0.3 and 0.5 mM oxalate increased OPN expression in cells; this was associated with time- and dose-dependent decreases in THP expression (Figure 6A). In the cell culture medium, we found that oxalate dose-dependently elevated OPN and THP release; increases in THP were also time dependent after treatment (Figure 6B). To estimate the degree of OPN and THP secretion, we calculated the secretion ratio by dividing their protein expressions in the culture medium by those in the cells. Compared with the control group, OPN secretion increased after 4 and 24 h of 0.3 mM oxalate treatment, whereas THP secretion was only increased at 24 h (Figure 6C). At the dose of 0.5 mM, the secretion ratio of both proteins and THP secretion were increased in a time-dependent manner. These results clearly indicated that oxalate increases OPN and THP in the cell medium.

Given that PKA signaling is known to affect OPN expression and tubular THP secretion [16,33], we then examined whether oxalate could activate PKA as part of its signaling transduction. Our results showed that oxalate dose-dependently increased intracellular cAMP levels (Figure 6D) and PKA activity after 24 h of treatment (Figure 6E).

### 3.7. PKA Inhibition or Hepsin Blockade Attenuates Oxalate-Mediated THP and OPN Secretion

**Figure 7:**
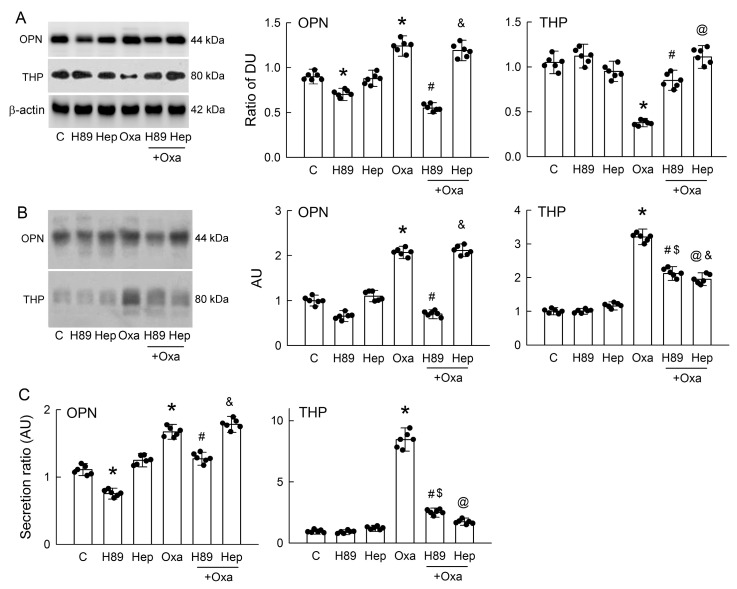
Blockade of PKA or hepsin attenuates oxalate-induced osteopontin (OPN) and Tamm–Horsfall protein (THP) secretion in cells. (**A**) Western blot analysis shows intracellular levels of OPN, THP, and β-actin in cells treated for 24 h with PBS (control, C), oxalate (Oxa, 0.5 mM), PKA inhibitor H89, or hepsin inhibitor hepIn-13 (Hep), either alone or in combination. The corresponding bar graphs (**right**) quantify OPN and THP relative to β-actin, expressed in density units (DU). (**B**) The representative blots of one experiment show OPN and THP expression in the culture medium after various treatments, as described above. The right bar graphs show the band density of OPN or THP and are expressed as arbitrary units (AU). (**C**) The ratio of OPN and THP secretion into the culture medium was estimated by dividing by the protein expression in cells and is expressed as AU. The scatter dots in the bar graphs show the data distribution. * *p* < 0.05, oxalate or H89 vs. control. ^#^ *p* < 0.05, H89 + Oxa vs. Oxa. ^@^ *p* < 0.05, Hep + Oxa vs. Oxa. ^$^ *p* < 0.05, H89 + Oxa vs. H89. ^&^ *p* < 0.05, Hep + Oxa vs. Hep.

Previous studies have shown that activation of PKA increases THP secretion via the effect of serine protease hepsin [15,16]. We therefore aimed to examine whether oxalate-mediated OPN and THP secretion is related to PKA and hepsin. Our results showed that the inhibition of PKA activity by a specific blocker H89 significantly attenuated OPN expression in cells without oxalate treatment (Figure 7A). H89 also markedly attenuated increased OPN expression caused by 0.5 mM of oxalate to a level lower than that of the controls. The blockade of hepsin by hepIn-13 (Hep) had no effect on OPN expression in cells with or without oxalate treatment. For THP, treatment of H89 alone showed no effect on its expression but significantly reversed oxalate-induced THP downregulation. Treatment of Hep alone also had no effect on THP expression. Similarly to H89, Hep significantly reversed oxalate-induced decreases in THP expression to a level similar to that in the control group.

In the cell culture medium, H89 alone slightly but insignificantly decreased OPN levels in the control cells; however, it completely abrogated increased levels of OPN by oxalate (Figure 7B). Treatment with Hep showed no effect on increased OPN levels in the oxalate-treated cells. H89 alone had no effect on THP expression but partially attenuated oxalate-mediated increases in THP expression. Treatment with Hep in the oxalate-treated cells significantly attenuated the THP upregulation that was induced by oxalate.

Estimating the secretion ratio of these two proteins, our results showed that treatment with H89 alone or in combination with oxalate lowered OPN secretion (Figure 7C). Treatment with Hep, however, had no effect on OPN secretion in the control cells or increased OPN secretion in the oxalate-treated cells. Interestingly, Hep completely reversed the increased THP secretion induced by oxalate. These results suggest that increased PKA or hepsin activity induced oxalate-mediated tubular cell secretion of OPN and THP in a different way.

### 3.8. H_2_S Donors Attenuate CaOx Crystal Formation and Protect Tubular Cell Against Oxalate

**Figure 8:**
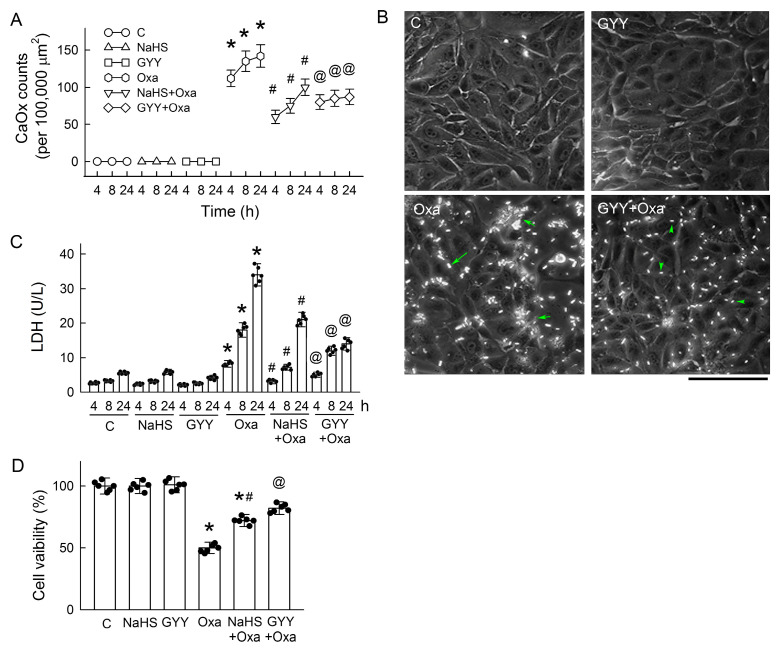
H_2_S donors attenuate oxalate-mediated CaOx crystal formation and cytotoxicity. (**A**) The formation of calcium oxalate (CaOx) crystals was assessed at 4, 8, and 24 h following treatment with 0.5 mM oxalate (Oxa) in the presence or absence of the H_2_S donors sodium hydrosulfide (NaHS) and GYY4137 (GYY). Both compounds reduced CaOx crystallization over time. (**B**) The representative bright-field images show the CaOx crystal formation in groups under 200× after 24 h of treatment. A long arrow in green indicates the typical long shape of a crystal (hexagonal lozenges). Short arrows in green indicate crystals clustering together. Arrow heads in green indicate that the crystal length was reduced by GYY, with a more spherical shape. (**C**) Oxalate-induced cytotoxicity, measured by lactate dehydrogenase (LDH) release, was attenuated by both NaHS and GYY. (**D**) The MTT assay results show improved cell viability after 24 h of treatment with H_2_S donors in oxalate-challenged cells. Each bar graph includes individual replicate values (N = 6). * *p* < 0.05, oxalate vs. control at the same time point. ^#^ *p* < 0.05, NaHS + Oxa vs. NaHS at the same time point. ^@^ *p* < 0.05, GYY + Oxa vs. GYY at the same time point.

We then supplied H_2_S donors to investigate the effect of H_2_S against oxalate-induced tubular damage and CaOx crystal formation. NaHS and GYY significantly attenuated oxalate-mediated increases in CaOx count in a time-dependent manner (Figure 8A). In our culture system, most oxalate-induced CaOx crystals appeared to have a long and narrow shape, similar to hexagonal lozenges (long arrow in Figure 8B); a few crystals were spherical in shape, and they were occasionally aggregated into clusters after 24 h of treatment (short arrow in Figure 8B). Treatment with GYY alone had no effect on the cell morphology. Interestingly, GYY not only reduced the number of CaOx crystals but also shortened the length of crystals (arrowhead in Figure 8B) and resulted in less aggregation of the crystals. Both H_2_S donors ameliorated oxalate-mediated tubular cell damage by reducing LDH release (Figure 8C) and increasing cell viability (Figure 8D), with a prominent effect found in treatment with GYY.

### 3.9. Exogenous H_2_S Supplement Attenuates OPN and THP Secretion in Tubular Cells

**Figure 9:**
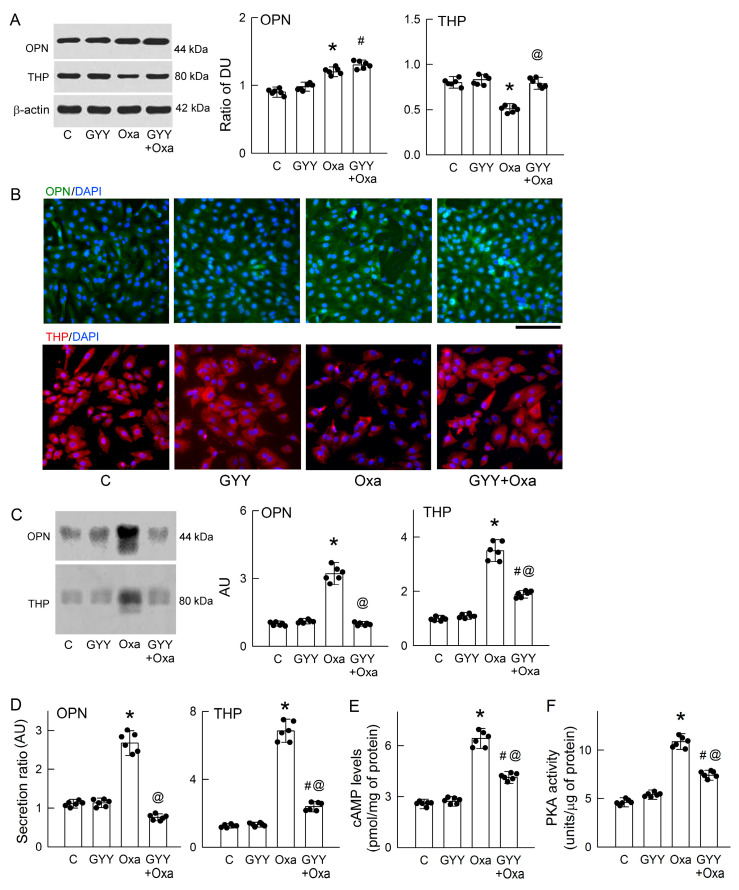
Exogenous H_2_S supplementation attenuates OPN and THP secretion and PKA activity caused by oxalate. (**A**) Western blot analysis reveals the intracellular expression of osteopontin (OPN), Tamm–Horsfall protein (THP), and β-actin in MDCK cells treated with PBS (control, C), 0.5 mM oxalate, or the H_2_S donor GYY4137 (GYY), alone or in combination for 24 h. Bar graphs (**right**) show the band density ratios of OPN and THP to β-actin, expressed as density units (DU). (**B**) The representative images show the immunoreactivity for OPN (green color) and for THP (red color) in cells with various treatments. Nuclei were counterstained using 4′,6-diamidino-2-phenylindole (blue color). The black horizontal bar = 50 μm. (**C**) The representative blots of one experiment show OPN and THP expression in the culture medium after various treatments. The right bar graphs show the band densities of OPN or THP, which are expressed as arbitrary units (AU). (**D**) The ratio of OPN and THP secretion into the culture medium was estimated by dividing by the protein expression in cells and is expressed as AU. (**E**) The cAMP level was determined in the cells in the groups. (**F**) PKA activity was determined in the cells in the groups. N = 6 experiments were performed in each group. The scatter dots in the bar graphs show the data distribution * *p* < 0.05, oxalate vs. control. ^#^ *p* < 0.05, GYY + Oxa vs. GYY. ^@^ *p* < 0.05, GYY + Oxa vs. Oxa.

The anticrystallization effect of GYY was further to test whether it may affect OPN and THP release in MDCK cells, as seen in our hyperoxaluric rats. Treatment with GYY did not affect oxalate-mediated increases in intracellular OPN expression; however, it reversed the reduction in THP expression caused by oxalate (Figure 9A). Compared with the control group, oxalate increased OPN expression, which was mainly distributed in the cytoplasm and occasionally present in the nucleus (upper parts in Figure 9B). GYY alone showed no significant effect on OPN expression. In the cells co-treated with GYY and oxalate, an increase in OPN expression was not only observed in the cytoplasm but also in the nucleus. Most THP was distributed in the cytoplasm of the control cells (lower parts in Figure 9B). GYY alone had no effect on THP expression, but it prevented the oxalate-induced loss of THP expression in the cytoplasm. In the culture medium, we found that GYY completely abolished oxalate-enhanced OPN release, and it partially attenuated oxalate-induced increase in THP release (Figure 9C).

We further explored the extent of OPN and THP secretion and found that GYY attenuated oxalate-induced increase in OPN and THP secretion when compared with the control group (Figure 9D). GYY alone had no effect on their secretion. We then investigated whether the effect was related to the cAMP/PKA pathway and found that GYY significantly reduced oxalate-mediated increases in intracellular cAMP levels (Figure 9E) and PKA activity (Figure 9F).

## 4. Discussion

**Figure 10:**
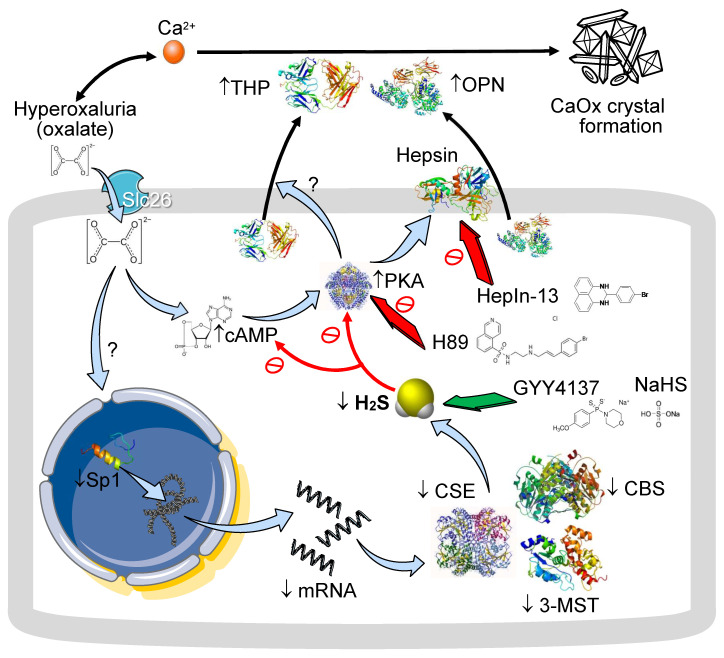
Mechanisms of H_2_S supplementation in preventing oxalate-induced tubular cell injury and calcium oxalate (CaOx) crystal formation and tubular cell injury. After oxalate (caused by hyperoxaluria) enters the renal tubular cells, it reduces the expression and activity of the transcription factor specificity protein 1 (Sp1) through an unknown mechanism, resulting in decreases in both mRNA and protein levels of the H_2_S-producing enzymes as cystathionine β-synthase (CBS), cystathionine γ-lyase (CSE), and 3-mercaptopyruvate sulfotransferase (3-MST), leading to a decrease in the formation of H_2_S. Deficiency in H_2_S results in tubular cell damage and calcium oxalate (CaOx) crystal formation in response to oxalate, and this can be attenuated by the H_2_S donors sodium hydrosulfate (NaHS) and GYY4137 (GYY). Moreover, oxalate treatment increases cyclic adenosine monophosphate (cAMP) and activates protein kinase A (PKA) to increase OPN excretion via an unexplored mechanism and probably enhance THP secretion via the effect of serine protease hepsin. The blockade of PKA by H89 attenuates both OPN and THP secretion. Tubular THP secretion also reduces the inhibition of hepsin by hepsIn-13. These effects are associated with the direct effect of H_2_S preventing CaOx crystal formation and tubular cell injury caused by hyperoxaluria. Black arrows indicate increases (upward) or decreases (downward). Red symbols (*Ө*) indicate inhibitory effects induced by H_2_S, H89, or hepsIn-13. Arrows in red curves indicate inhibitory effects of H_2_S. Light blue arrows indicate downstream target effects. Red widened flat arrows indicate inhibitory effects after drug treatment. Green widened flat arrows indicate exogenous H_2_S supplementation. Question marks indicate the mechanism not investigated in this study.

As shown in the scheme summarized in Figure 10, hyperoxaluria, either induced by feeding rats a HP diet in vivo or via direct oxalate treatment in renal tubular cells in vitro, suppresses H_2_S formation by lowering Sp1 activity and its downstream effect on the expression of H_2_S-producing enzymes such as CBS, CSE, and 3-MST, at both the mRNA and protein levels. H_2_S deficiency leads to tubular injury and CaOx crystal formation, deposition, and aggregation in response to hyperoxaluria or oxalate exposure, a process that can be attenuated by H_2_S donors such as GYY or NaHS. Oxalate triggers increases in cAMP and PKA activation, which in turn increases OPN and THP secretion, whereas the PKA inhibitor H89 abolishes their secretion. Blockade of the serine protease hepsin by HepIn-13 also reduces oxalate-induced THP secretion. Supplementation with H_2_S by GYY attenuates oxalate-induced increases in PKA activity and tubular OPN and THP secretion. These findings support the hypothesis and highlight that H_2_S is an anticrystallization molecule with the therapeutic potential for preventing kidney stone formation and tubular damage in hyperoxaluria.

In the hyperoxaluric rats and oxalate-treated tubular cells, we observed typical manifestations of oxalate nephropathy, which was associated with renal H_2_S deficiency (Figure 1 and Figure 5). Oxidative stress is a well-recognized driver of hyperoxaluria-induced tubular injury and crystal nephropathy, as previously reported in experimental models and in patients [7]. Hyperoxaluria-mediated oxidative stress not only increases the enzymatic activity of oxidases such as NADPH oxidase (gp91phox) and xanthine oxidase but also leads to reduced antioxidant defenses, including the activities of superoxide dismutase (SOD), glutathione peroxidase (GPx), and glutathione reductase (GR) [29,34]. The resulting redox imbalance primes the tubular cell injury for further triggering crystal adhesion and inflammation [7]. However, the mechanism of how hyperoxaluria impairs antioxidant protein activity is still unclear. In addition to its direct antioxidant effect, H_2_S is known to increase the activities of SOD, catalase, GPx, and GR, as well as GSH content, to enhance antioxidant defense [35,36]. Deficiency in H_2_S probably is the last piece of the puzzle, showing why the loss of antioxidant protein defense in oxidative stress is caused by hyperoxaluria.

H_2_S deficiency was found after 3 days of hyperoxaluric induction, a time point with little CaOx deposition in kidneys (Figure 1); this indicates that chronic hyperoxaluria can initiate decreases in H_2_S formation independent of the presence of CaOx crystals. H_2_S decreased thereafter as the severity of hyperoxaluria increased. Unlike the rat model, MDCK cells exposed to oxalate showed that H_2_S deficiency was concurrent with cell damage and CaOx crystal formation (Figure 5A–C). We then showed the impaired H_2_S production is related to decreased Sp1 activity and downstream H_2_S-producing enzyme expression in both models (Figure 2, Figure 3 and Figure 5). Previous studies have reported that about 75% of renal cells and 87% of endothelial cells in total express H_2_S-producing enzymes, making the kidney a rich source of endogenous H_2_S production [37]. Our results showed that most of the H_2_S-producing enzymes were distributed in renal tubules of the control kidneys, while these were attenuated in the hyperoxaluric kidneys (Figure 3). Our in vitro study further confirmed the direct effect of oxalate or CaOx on decreased H_2_S-producing enzyme expression in renal tubular cells (Figure 5F,G). As the kidney is a highly blood-perfused organ, receiving approximately 20–25% of cardiac output, we propose that the loss of H_2_S-generating enzymes in renal tubules profoundly impacts systemic H_2_S levels, as we demonstrated decreased H_2_S levels in the renal venous blood of hyperoxaluric rats (Figure 1G). This is consistent with previous reports showing that deficiencies in H_2_S-producing enzymes and markedly reduced plasma H_2_S levels correlate with the severity of kidney diseases in human patients and experimental animals [37].

Given the reduction in H_2_S levels, it is important to further investigate the upstream signals that regulate H_2_S-producing enzyme expression. As a transcription factor, zinc finger structures in Sp1 make it specifically bind to GC-rich regions in the promoter regions of target genes for the induction of cell differentiation, proliferation, and growth [38]. Decreases in the DNA-binding activity of Sp1, even when its protein levels are normal, can lead to decreased renal CBS expression and contribute to kidney injuries induced by I/R [39]. Here, we showed consistent results in another kidney disease model, indicating that hyperoxaluria and oxalate markedly suppressed Sp1 protein levels and activities (Figure 2D,E and Figure 5D,E). Interestingly, an increase in Sp1 expression has been shown to protect kidneys from I/R injury [40]. Although the present study did not directly investigate how hyperoxaluria and oxalate impair Sp1 expression and activity, previous studies have provided several upstream mechanisms that may contribute to altered Sp1 activity. First, increased intracellular calcium concentration ([Ca^2+^]_i_) can induce phosphorylation or conformational changes in Sp1, thereby reducing its DNA-binding affinity and altering downstream gene transcription in neuronal and epithelial cells [41,42]. Interestingly, oxalate is known to increase [Ca^2+^]_i_ from intracellular stores, L-type and T-type calcium channels, and transient receptor potential vanilloid 1 channels in neuroblastoma, smooth muscle, neuronal, and renal tubular cells [43,44]. Second, stress-activated protein kinase/mitogen-activated protein kinase (SAPK/MAPK), including extracellular signal-regulated kinase (ERK), p38-MAPK, and c-Jun N-terminal kinase (JNK), are known to regulate Sp1 activity. In rat kidneys subjected to I/R, ERK suppresses CBS gene expression by enhancing Sp1 phosphorylation and reducing its transcriptional activity [39]. Mechanical stress activated p38-MAPK, which increased Sp1 phosphorylation at specific threonine residues and enhanced the expression of Sp1-dependent genes in Rat-2 cells and human gingival fibroblasts [45]. In the human monocyte cell line, a previous study showed that p38-MAPK regulates lipopolysaccharide-induced activation of Sp1 in interleukin-10 formation [46]. Moreover, cumulated studies have revealed that JNK activation is necessary to phosphorylate Sp1; increased Sp1 phosphorylation, however, reduces Sp1 binding to DNA [47,48]. Interestingly, increased ERK activity in hyperoxaluric kidneys is involved in oxidative stress and CaOx crystal formation [49]. The exposure of oxalate to renal tubular cells (LLC-PK_1_) selectively activates p38-MAPK and JNK signaling pathways, indicating that p38-MAPK/JNK activity is essential for the effects of oxalate on tubular cell function [50]. The activation of p38-MAPK also contributes to tight junction disruption in distal renal tubular cells caused by treatment with CaOx monohydrate crystals [51]. Finally, hyperoxaluria and CaOx stones are known to induce ROS production in oxidative stress to promote kidney damage [52]. A previous study showed that treatment with hydrogen peroxide increased the methylation of Sp1 and repressed Sp1 transcriptional activity [53]. Further studies are required to explore the novel role of calcium, MAPKs, and ROS in Sp1 function in terms of H_2_S production in hyperoxaluria.

OPN and THP are two urinary macromolecules that play a significant role in kidney stone formation. However, the role of OPN or THP in promoting or inhibiting nephrolithiasis is currently under debate [54,55]. Our results showed that urinary OPN and THP levels and their secretions by renal tubular cells were markedly elevated in hyperoxaluric rats and in oxalate-treated cells (Figure 2 and Figure 6). The possible reason why hyperoxaluria and oxalate facilitate OPN and THP secretion by renal epithelium is that they act as anticrystallization agents, as previously reported [52]. In an in vitro study, OPN induced changes in CaOx crystalline from monohydrate (COM) to dehydrate (COD) form and reduced CaOx attachment to tubular cell surfaces [56]. THP exhibits a strong affinity, specifically binding with calcium; this inhibits COM growth, aggregation, and crystal–cell adhesion [54]. In the present study, hyperoxaluria induced urinary supersaturation and the direct treatment of oxalate induced remarkable CaOx formation (Figure 1C and Figure 5A); this stress makes renal tubular cells secrete more OPN and THP and probably induces the formation of less dense and more soluble COD crystals for elimination by urinary excretion. Interestingly, enhanced OPN and THP secretion after hyperoxaluric induction depletes their contents in renal tissues over time (Figure 2B). The intracellular content of THP, but not OPN, in oxalate-treated MDCK cells also showed a time-dependent decrease similar to that detected in vivo (Figure 6A). In renal tubular cells, OPN and THP play crucial roles in maintaining epithelial function: OPN is responsible for cell adhesion and survival, whereas THP is responsible for maintaining apical polarity and the barrier function of tubular cells [19,57]. Intracellular depletion of OPN and THP may therefore impair cytoskeleton organization and tight junction integrity, leading to epithelial fragility [13,14], which is especially vulnerable to hyperoxaluria. Cellular debris resulting from this epithelial function further serves as the seed in tubular lumen for more crystal formation, aggregation, and growth in a vicious cycle. Our in vitro results showed differences in cellular OPN and THP expression after oxalate treatment, i.e., OPN being upregulated and THP downregulated (Figure 6A). Indeed, this discrepancy is consistent with previous findings that the mRNA levels of OPN and THP gradually increased and decreased, respectively, in hyperoxaluric rats after 14 and 28 days of induction and in the OPN/THP-knockout mice [32,58]. The previous study suggests that THP serves as a constitutive inhibitor of crystallization while OPN may act as an inducible inhibitor [32]. The increased OPN expression in the MDCK cells is similar to a recent study examining the treatment of renal tubular cells NRK-52E with 1 mM oxalate [59]. Although the increase in OPN caused by oxalate or CaOx crystals facilitates crystal adhesion to damaged epithelial cells for further retention and growth [60], this intracellular increment may support cells against further damage due to oxalate or CaOx crystals because OPN plays a crucial role in maintaining epithelial integrity.

Previous studies have shown that oxalate or CaOx crystals can activate various intracellular protein kinase signaling, such as protein kinase B (Akt), protein kinase C, MAPKs, and receptor-interacting serine/threonine-protein kinase 3, to induce cellular adaptive response in renal tubular cells [61,62,63,64]. Interestingly, the present results demonstrated for the first time that oxalate treatment in the presence of CaOx crystals enhanced the cAMP/PKA signaling pathway in MDCK cells (Figure 6). In fact, PKA activity plays an important role in the regulation of renal tubular function. For example, PKA interacts with other signaling proteins such as MAPKs to regulate actin dynamics for apical protein sorting and the vesicle trafficking of aquaporin-2 via the effect of G protein-coupled receptors (GPCRs) after stimulation by vasopressin in the mouse cortical collecting duct cells [65]. In COM-treated renal tubular cells and hyperoxaluric rats, a previous study also showed that PKA can be activated by another GPCR, calcium-sensing receptor, thereby affecting claudin14 expression, possibly due to calcium reabsorption [66]. In the present study, the inhibition of PKA by H89 attenuated cellular OPN contents not only in the oxalate-treated cells but also in the control cells (Figure 7A). The direct effect of PKA on OPN expression is further supported by a previous study showing that H89 reduces the increased mRNA levels of OPN after adrenomedullin treatment in vascular smooth muscle cells [33]. More than PKA, the activation of ERK-MAPK after genistein treatment was shown to reduce the amount of OPN secreted in metastatic cancer cells [67]. Unlike OPN, the PKA inhibitor H89 exhibited the opposite effects on THP by increasing its intracellular content, which was reduced by oxalate (Figure 7A). This can be explained by the attenuation of oxalate/cAMP/PKA-triggered THP secretion, thereby retaining THP inside cells to further support tubular cell function in stress. The blockade of PKA by H89 had the same effect on reducing the oxalate-mediated secretion of both OPN and THP (Figure 7B,C). Furthermore, the inhibition of the serine protease hepsin with a specific blocker hepIn-13 had no effect on the intracellular and secreted OPN expression by oxalate, indicating that OPN secretion by tubular cells is independent of hepsin. However, hepIn-13 markedly attenuated THP secretion by oxalate (Figure 7A–C). Previous studies have revealed that the secretion of OPN and THP depends on the enzymatic cleavage activity of serine proteases [15]. OPN could be cleaved by thrombin, matrix metalloproteinases, plasmin, and cathepsin D to generate OPN variants with different activities for specific biological functions [68]. After proteolytic cleavage via hepsin, THP may exert its function as an extracellular matrix and lead to the conformational activation of a Zona Pellucida polymerization domain, as found in the egg coat and the inner ear tectorial membrane [15]. These results may explain why hepIn-13 only functions to block oxalate-induced THP secretion but not OPN secretion. Furthermore, a recent study demonstrated that vasopressin-mediated PKA activation acts as a physiological stimulus of urinary THP secretion in mouse kidneys and in polarized MDCK cells [16]. Further studies are required to see whether PKA may target these serine proteases directly to enhance their enzymatic cleavage activities in terms of tubular OPN and THP secretion.

The restoration of H_2_S via treatment with H_2_S donors NaHS or GYY not only alleviated tubular injury and the crystal burden but also attenuated OPN and THP secretion in both in vivo and in vitro hyperoxaluric models (Figure 4, Figure 8 and Figure 9). In hyperoxaluric rats, these effects occurred without affecting the urinary excretion of oxalate, suggesting that GYY-mediated tubuloprotection and anticrystallization are downstream of oxalate. The antilithiatic effect of H_2_S liberated from GYY in rats can exclude its effect on urine pH, because H_2_S dissociates into HS^−^ and S_2_^−^ ions and makes the aqueous solution more acidic. An acidic pH in urine favors CaOx crystal formation [69]. Therefore, the beneficial effect of H_2_S seen in this study depends on its cytoprotection. In renal tubular cells, both NaHS and GYY significantly decreased oxalate-induced spontaneous CaOx crystal formation, but with different efficacies, as NaHS demonstrates a steeper change than GYY (Figure 8A). Based on their pharmacokinetic profiles for H_2_S liberation, NaHS is a fast-releasing chemical and exerts a rapid effect, while GYY is a slow-releasing compound that provides a sustained effect, as evidenced by reduced cell damage and increased viability after co-treatment (Figure 8C,D) [70,71]. Interestingly, the typical crystal shape of CaOx in our culture system was a long cylindrical shape, similar to the picket fence shape found in ethylene glycol poisoning (Figure 8B), validating the feasibility of using this in vitro system as a platform to explore phenotyping changes in the kinetics of CaOx formation. The beneficial effects of GYY against oxalate were confirmed not only in terms of the crystal number but also in crystal length and the degree of clustering.

In addition to the cytoplasm, our immunofluorescent labeling showed that OPN was also present in the nucleus of control cells, and nuclear distribution increased after oxalate or GYY treatment (Figure 9B). The nuclear localization of OPN in MDCK cells is similar to that in embryonic kidney 293 cells [68]. The nuclear localization of OPN is correlated with chromatin condensation during cell division [68]. This may facilitate cell recovery from toxic oxalate after treatment or GYY-mediated cytoprotection. For THP, oxalate or GYY mainly affected its cytoplasmic distribution. Moreover, GYY significantly decreased oxalate-induced OPN and THP release from tubular cells (Figure 9C,D), indicating that the changes in OPN and THP excretion seen in our hyperoxaluric rats originated from the renal tubules. A previous study showed that H_2_S inhibited adenylyl cyclase to reduce cAMP levels in the constriction of rat cerebral basilar arteries [72]. H_2_S also attenuated the adenylyl cyclase/cAMP pathway to impede the development of opioid dependence [73]. Moreover, H_2_S inhibited the effect of the PKA activator forskolin on renin degranulation from HMC-1.1 mast cells by lowering intracellular cAMP levels [74]. Although we did not measure changes in adenylyl cyclase activity and intracellular cAMP levels in the treated MDCK cells, our results showed that the H_2_S donor GYY directly reduced intracellular cAMP levels and PKA activity in oxalate-treated cells (Figure 9E,F). As a second messenger, cAMP formed by any form of adenylyl cyclase is responsible for the activation of PKA [75]. PKA is generally associated with beneficial cellular processes; however, excessive or dysregulated PKA activity can induce oxidative stress under certain conditions. A previous study has shown that PKA is involved in oxidative stress in cells exposed to hypoxia and in myocardial tissues after I/R, leading to mitochondrial dysfunction by increasing the activity and phosphorylation of cytochrome c oxidase, thereby releasing ROS [76]. Since oxidative stress is a central mechanism in oxalate-mediated renal tubular damage, reducing the role of PKA (as we demonstrated using H_2_S) may provide a novel therapy in crystal nephropathy. The protective effect of H_2_S is not limited to PKA; it also protects cells against oxidative stress by increasing intracellular GSH levels, scavenging ROS/RNS, maintaining the mitochondrial redox balance for ATP production, reducing inflammation, sulfurylating signaling proteins, enhancing nitric oxide signaling, and preventing apoptosis [77,78]. Here, we showed another potential mechanism for H_2_S-mediated tubuloprotection against oxalate/cAMP/PKA pathway: namely, preserving the renal expression of OPN and THP by reducing tubular wasting.

This study has some limitations. Our in vitro observations were obtained from MDCK cells, which typically have a distal tubule origin. Whether H_2_S provides the same protective effect to other tubular segments such as the proximal tubule requires further study. Furthermore, we observed cellular changes in MDCK cells after exposure to oxalate for up to 24 h, a short induction time that may not fully reflect the long-term pathophysiological changes observed in the chronic hyperoxaluric state. Experimental models other than HP-induced chronic hyperoxaluria and genetic animal models with overexpression of H_2_S-producing enzymes are also required to ensure consistent observation of the antilithiatic effects of H_2_S and serve as a basis for translation to clinical trials. Moreover, while we used histological staining for the semi-quantitative scoring of CaOx crystal deposition, more advanced imaging techniques such as micro-computed tomography (micro-CT) may offer three-dimensional precision in detecting the surface and internal composition of kidney stones and the effectiveness of treatment.

## 5. Conclusions

Our results clearly reveal that H_2_S deficiency contributes to tubular damage and CaOx crystal formation in both a rat hyperoxaluric model and oxalate-exposed cultured renal tubular cells. The decline in H_2_S production is linked to decreased expression of its producing enzymes—CBS, CSE, and 3-MST—and to impaired DNA-binding activity of their upstream regulator, Sp1. Increased cAMP/PKA signaling induces the excessive tubular secretion of two anticrystallization molecules, OPN and THP, and depletes their renal contents, which are involved in hyperoxaluria/oxalate-induced tubular damage and CaOx crystal formation. The replenishment of H_2_S attenuates cAMP levels and PKA activity, reversing these effects, representing a promising approach to treating kidney stones.

## Figures and Tables

**Table 1:** List of primer sequences used for real-time quantitative polymerase chain reaction (RT-qPCR).

Gene	GenBank Accession Number	Primer Sequence
CBS	XM_039080137	5′-TAGACGGCAGAGCCTTTCGA-3′ (forward) 5′-AATCCCCGGCCGTAGAAC-3′ (reverse)
CSE	NM_017074	5′-ACACTTCAGGAATGGGATGG-3′ (forward) 5′-TGAGCATGCTGCAGAGTACC-3′ (reverse)
3-MST	NM_001013440	5′-CTGGGAAACGGGGAGCG-3′ (forward) 5′-GCTCGGAAAAGTTGCGGG-3′ (reverse)
GAPDH	XM_039097338	5′-TTAGCACCCCTGGCCAAGG-3′ (forward) 5′-CTTACTCCTTGGAGGCCATG-3′ (reverse)

CBS, cystathionine β-synthase; CSE, cystathionine γ-lyase; 3-MST, 3-mercaptopyruvate sulfurtransferase; GADPH, glyceraldehyde 3-phosphate dehydrogenase.

## Data Availability

The data presented in this study are available on request from the corresponding author due to institutional data protection and ethical considerations related to animal research.

## Abbreviations

3-MST	3-mercaptopyruvate sulfurtransferase
AP(CaOx)	Ion activity product of calcium oxalate
[Ca^2+^]_i_	Intracellular calcium ion concentration
CaOx	Calcium oxalate
cAMP	Cyclic adenosine monophosphate
CBS	Cystathionine β-synthase
CSE	Cystathionine γ-lyase
COD	Calcium oxalate dihydrate
COM	Calcium oxalate monohydrate
DAPI	4′,6-diamidino-2-phenylindole
ERK	Extracellular signal-regulated kinase
GAPDH	Glyceraldehyde 3-phosphate dehydrogenase
GYY	GYY4137
GPCR	G protein-coupled receptor
GPx	Glutathione peroxidase
GR	Glutathione reductase
H_2_S	Hydrogen sulfide
H89	PKA inhibitor
Hep	HepIn-13
HP	Hydroxy-L-proline
HRP	Horseradish peroxidase
I/R	Ischemia/reperfusion
JNK	c-Jun N-terminal kinase
KIM-1	Kidney injury molecule-1
LDH	Lactate dehydrogenase
LLC-PK_1_	Lilly Laboratories cell-porcine kidney 1
MAPK	Mitogen-activated protein kinase
MDCK	Madin–Darby Canine Kidney
MTT	3-(4,5)-2,5-diphenyltetrazolium bromide
NADPH	Reduced nicotinamide adenine dinucleotide phosphate
NaHS	Sodium hydrosulfide
NO	Nitric oxide
OPN	Osteopontin
PBS	Phosphate-buffered saline
PKA	Protein kinase A
qPCR	Quantitative real-time polymerase chain reaction
RNS	Reactive nitrogen species
ROS	Reactive oxygen species
SOD	Superoxide dismutase
Sp1	Specificity protein 1
THP	Tamm–Horsfall protein
